# Some of the Effects of Tobacco

**Published:** 1862-09

**Authors:** P. J. Farnsworth

**Affiliations:** Lyons, Iowa


### SOME OF THE EFFECTS OF TOBACCO.

By P. J. FARNSWORTH, M.D., Lyons, Iowa.

Mr. L., aged 35, a plasterer by trade, called at my office Dec. 12, 1861, complaining of sore throat, together with partial anæsthesia of his fingers, toes, and end of his tongue. According to the statement he made to me, he had always been regular and temperate in his habits. He had a wife and four children. He was rather thin, and the noticeable feature about him was a partial anæmic or cachectic look. His appetite was good. As the numbness was not troublesome I thought it might be temporary, and did not at that time attempt any treatment. There was redness of the fauces, and a slight enlargement of the tonsils, for which I used a topical application of a weak solution of nitrate of silver. Dec. 20th, he came again to the office for another application to the throat, complaining still more of numbness. I was then led to make a more careful examination of his case, with a view to find some definate cause for these nervous phenomena. I could find no tenderness either superficial or deep-seated. He had never been troubled with headache, had had no pain in the lumbar region. There was something in his look that suggested Bright's disease, and I accordingly examined the urine rigidly, but found nothing abnormal. I gave him some simple bitters, with a preparation of iron, and a blister to apply to the spine, between the shoulders, commencing the treatment with a dose of compound cathartic pills.

Jan. 1st, 1862, L. was brought to the office, having previously been able to walk (the distance of five miles). Now the ride was too much for his strength. The numbness was increasing, his appetite was somewhat inordinate, and the tendency to constipation, which previously existed, had increased; he had lost flesh. I again examined the urine without detecting anything. Thinking it might be a miasmatic poisoning, ordered three grains of quinine three times a day, together with a laxative pill to be taken every night. On the 12th of January I was sent for, as his strength had failed, and he was confined to the house. At this time I asked a neighboring practitioner to visit him in consultation. The numbness now seemed pretty general; his eyesight was somewhat affected. The heart beat somewhat irregularly, with a slight anæmic murmur. The tongue was slightly coated. The generative organs were participating in the general weakness; had no erections or sexual desires. Gave him tinct. fer. mur. gutt. x. three times a day, and agreed to the reapplication of the blister to the spine.

I called Jan. 15th, and found the patient in bed, very much dejected, and *smoking*. Having heard us discuss the propriety of blistering, he had applied the blisters to the whole spine, and to the hips. He felt them but little, and was scarcely conscious of their presence, but they had produced an immense drain on the system, and he had failed very fast. I questioned him now about his smoking, and found that he had considered it so small a matter as not to mention it, but that for several years he had smoked the coarsest kind of tobacco in the foulest kind of a pipe, during all his waking hours. On further examination into his case, I was convinced that it was one of poisoning by nicotine. The want of feeling was now general; the motor powers were active enough; he could hold his knife in his hand firmly when his eyes were directly on it, but if he looked away the knife dropped. He walked with difficulty, because he must needs look where his feet fell, as he could not feel them touch the ground. I advised him to stop his smoking at once, and ordered an ounce of brandy and potass. iod. gr. v. three times a day, friction to the surface, and a Dover's powder at night, as he had become very wakeful.

On the third day after I found the patient somewhat improved, but now alarmed at the quantity and quality of his urine; it was very highly charged with a red sediment, which proved to be urea, urate of ammonia, and triple phosphates; with a distinct odor of nicotine. The Dover's powder acted as a diaphoretic, conjoined with the friction; and he complained of a fetid sweat. His wife said that now he had discarded the pipe, the fumes of it were exuding through his skin. Continued the brandy and the potass iod. and gave him tinct. fer. mur. gutt. xx. three times a day. On the 22d inst., I found the patient improving, the urine becoming more natural, but the perspiration was profuse at night, having the same nicotine odor. Sensibility did not return as fast as the strength; he was obliged to use his reason about cold and heat, and in regard to taking food. Cold was scarcely felt, and but little taste was left. Even his exercise had to be regulated by his judgment, and not by his feelings. I continued the tinct. fer. mur. and the potass iod., giving an occasional dose of sulph. magnesia, to move the bowels.

*Jan.* 29.—The patient still improving, the numbness going of in the same manner that it had come on—passing downwards towards the extremities. I continued the iron, ordered a nourishing diet, the bathing every evening with friction, and an occasional Dover powder.

From that time until March he continued to improve, the numbness having passed to the ends of the fingers and toes, and the end of the tongue. He was then able to walk the five miles to see me; the waxy look had passed off; and the look of health returned. At this time, for experiment sake, he tried to smoke a cigar. It produced nausea and all the feelings that tobacco does to a novice; together with a return of the numbness of the fingers, and a smarting of the fauces.

I consider this as an illustration of the effects of the slow introduction of tobacco poison into the system. I have been unable to find any such effect ascribed to it in any work I have at hand. Dr. Prout, quoted by Pereira, says, "It disorders the assimilating functions in general; but particularly, as I believe, the assimilation, of the saccharine principle. I have never indeed been able to trace the development of oxalic acid to the use of tobacco; but that some analogous and equally poisonous principle (probably of an acid nature) is generated in certain individuals by its abuse, is evident from the cachectic looks, and from the darkened, often greenish-yellow tint of their blood."

Pereira says, "I am not acquainted with any well ascertained ill effects resulting from the habitual use of smoking."

He is probably a lover of the "weed" himself, or had not made close observation of the smokers around him, for it is my opinion that scarcely one in five but that is injured by tobacco. It acts as a nervous sedative or depressant, causing an exhausted feeling at the stomach, and I have good reason to suppose that smoking is the great cause of the universal dyspepsia with which we are troubled.

It sometimes produces tremor of the nerves, and in many cases, instead of being a temporary sedative, destroys the tone of the nerves and produces languor and fretfulness, and often effects like those above described, though not in every case in so marked a degree. In a majority of smokers and chewers of tobacco, it acts like a violent poison, which it is, introduced slowly.—*Amer. Medical Times.*

